# Impact of APRF+ in Combination with Autogenous Fibroblasts on Release Growth Factors, Collagen, and Proliferation and Migration of Gingival Fibroblasts: An In Vitro Study

**DOI:** 10.3390/ma15030796

**Published:** 2022-01-21

**Authors:** Barbara Sterczała, Agnieszka Chwiłkowska, Urszula Szwedowicz, Magdalena Kobielarz, Bartłomiej Chwiłkowski, Marzena Dominiak

**Affiliations:** 1 Dental Surgery Department, Wroclaw Medical University, 50-425 Wroclaw, Poland; marzena.dominiak@umed.wroc.pl; 2 Department of Molecular and Cell Biology, Wroclaw Medical University, 50-556 Wroclaw, Poland; WF-26@umw.edu.pl; 3 Department of Mechanics, Materials and Biomedical Engineering, Wroclaw University of Science and Technology, 50-371 Wroclaw, Poland; magdalena.kobielarz@pwr.edu.pl; 4 Department of Applied Mathematics, Faculty of Pure and Applied Mathematics, Wroclaw University of Science and Technology, 50-370 Wroclaw, Poland; 249761@student.pwr.edu.pl

**Keywords:** A-PRF+, fibroblast culture, wound healing, VEGF, TGFβ2

## Abstract

The present study aimed to compare the action of advanced platelet-rich fibrin (A-PRF+) alone with the action of A-PRF+ combined with autologous gingival fibroblasts. The components released from A-PRF+ conditioned with autogenous fibroblasts that were quantified in the study were fibroblast growth factor (FGF), vascular endothelial growth factor (VEGF), trans-forming growth factor-beta1 and 2 (TGFβ1 and TGFβ2), and soluble collagen. A-PRF+ combined with fibroblasts demonstrated significantly higher values of released VEGF at every time point and, after 7 days, significantly higher values of released TGFβ2. A viability test after 72 h showed a significant increase in proliferation fibroblasts after exposition to the factors released from A-PRF+ combined with fibroblasts. Similarly, the degree of wound closure after 48 h was significantly higher for the factors released from A-RRF+ alone and the factors released from A-RRF+ combined with fibroblasts. These results imply that platelet-rich fibrin (PRF) enhanced with fibroblasts can be an alternative method of connective tissue transplantation.

## 1. Introduction

Platelet-rich fibrin (PRF) contains supraphysiological concentrations of growth factors that stimulate bone and soft tissue regeneration in a natural way [1]. The protocol of obtaining PRF of the second generation, introduced by Choukroun and colleagues [2], allows one to achieve material that is completely autologous and prepared without any anticoagulants or separators. PRF contains leukocytes, as well as biochemical components, such as growth factors (GFs); platelets; immunity promoters; and cytokines, including IL-1 β, IL-4,IL-6, and TNF- α [3,4], which stimulate the healing process.

Leukocytes and fibrinogen reduce the harmfulness of the hypermetabolic phase in the first phase of healing [5]. The strong network of a PRF clot consists of polymerized fibrin and chains of structural glycoproteins [4]. Due to its biomechanical properties, the membrane is easy to use clinically. It shows flexibility and elasticity, and it is easy to form. Currently, PRF is successfully used in modern periodontal regenerative stomatology [6], among other things, due to the ease of acquirement, the activity at every stage of soft-tissue healing, and the economic aspect [2,7,8,9].

Recent advances in medical sciences have led to the development of a new procedure to obtain various products of PRF, such as APRF+ [10]. The method, speed, and time of centrifugation of the venous blood taken from the patient greatly influence the composition of the clot: the number of platelets, leukocytes, and GFs [10]. If less force and a shorter time of centrifugation are used, more leukocytes, and thus monocytes, and macrophages are obtained, which, in turn, increases the number of precursor cells at the site of application; therefore, this corresponds to improved regenerative potential. A significantly increased level of released growth factors corresponds to the increase in the number of platelets, evenly distributed in the fibrin network [8,10,11,12,13]. Transforming growth factor-beta (TGFβ), vascular endothelial growth factor (VEGF), epidermal growth factor (EGF), platelet-derived growth factor (PDGF), and insulin-like growth factor (IGF) affect intracellular and intercellular communication and, thus, stimulate cell migration, adhesion, and proliferation at the wound site [12,14,15]. In turn, the fibrin present in the network stimulates a slower degradation of the network and delays the release of growth factors for 7–10 days, which is in contrast to PRP, where the growth factors are secreted within the first hour [16,17]. In addition, sufficiently large gaps in the scaffold of the APRF+ matrix allow neutrophils to penetrate it, which affects the functionality of the transplanted and local host cells in the regenerated tissue [8,13].

Therefore, APRF+ is used as a natural polymer in tissue engineering, and the available knowledge concerning its application allows us to state the validity of the use of A-PRF+ as a carrier for isolated autogenous fibroblasts for the augmentation of keratinized gingiva. Fibroblasts play a crucial role in three stages of tissue regeneration by releasing growth factors, which regulate the processes of intra- and extra-cellular metabolism, indirectly modulating the formation of a new extracellular matrix (EMC) [14,18,19]. The advantage of autogenous cell cultures is that they provide biomaterial for augmentation in the amount of determined tissue loss.

The present study aimed to determine whether the combination of A-PRF+ with autogenous fibroblasts would change the number of released components that are important in the context of the healing processes, including fibroblast growth factor (FGF); vascular endothelial growth factor (VEGF); transforming growth factor-beta1 and 2 (TGFβ1 and TGFβ2); and collagen, the main protein of the extracellular matrix, produced by fibroblasts. The impact of the released components on the proliferation of fibroblasts and their migration was analyzed. The motivation to conduct the present study is the evolution and enhanced methods of wound healing.

## 2. Materials and Methods

### 2.1. Cell Culture and A-PRF+-Based Matrices

Primary human gingival fibroblasts (HGFs) and A-PRF+ were obtained from six systematically healthy volunteer donors, following approval by the Ethics Committee of Wroclaw Medical University, Poland (No KB-434/2017). Samples of hard palatal and gingival tissues were collected in the amount of 1–2 mm^2^ from each donor and transported to the laboratory in the nutrient medium Dulbecco’s modified Eagle’s medium (DMEM, Sigma-Aldrich, Poznan, Poland) with the addition of 10% fetal calf serum (Gibco-ThermoFisher, Warsaw, Poland), penicillin (100 Ul/mL), streptomycin (0.1 mg/mL), and amphotericin B (0.1 mg/mL). Subsequently, fibroblasts were mechanically isolated and cultured according to the patented method described by Dominiak et al. [20]. The culture was carried out in a conventional DMEM culture medium in an incubator at 37 °C in a 5% CO_2_ atmosphere. The culture medium was changed twice a week. The cells reached a full monolayer after 5–7 days. After achieving a full monolayer of cells, four tubes of blood samples were collected in the amount of 10 mL from these same six volunteer donors. Next, A-PRF+ was obtained according to the procedure developed by Choukroun [11]. The blood samples without anticoagulant were centrifuged at 1300 rpm (200× *g*) for 8 min in a centrifuge machine PRF DuoTM (Process for PRF, Nice, France). The A-PRF+ clots were removed from the tubes and separated from the RBC base using sterilized scissors for further investigation.

### 2.2. Assessment of Growth Factor Release from Fibroblasts Alone, A-PRF+ Alone, and A-PRF+ with Fibroblasts

Primary human fibroblasts at a concentration of 4 × 10^5^ cells/mL were placed into a twelve-well dish with 1.5 mL of culture media (DMEM) and allowed to grow for 24 h. Then, the medium was removed, and the sterile-flattened A-PRF+ clots were placed (not less than within 1 h from production) in a well with fibroblasts and in an empty well, and there was one well that contained only fibroblasts. Fresh medium in the amount of 1.5 mL was added to each variant. At 1, 2, 3, and 7 days, 1.5 mL of culture media was collected, frozen at −20 °C, and replaced with 1.5 mL of fresh culture media. The content of soluble collagen, TGFβ1, TGFβ2, FGF1, and VEGF in the collected medium was investigated. The release of growth factors was quantified using the colorimetric test for collagen quantification and ELISA for the investigation of the remaining factors.

### 2.3. The Quantification of Growth Factors with Enzyme-Linked Immunosorbent Assay (ELISA)

To determine the amount of growth factors released from A-PRF+ alone, A-PRF+ with fibroblasts, and only fibroblasts alone at days 1, 2, 3, and 7, samples were investigated using ELISA. At the desired time points, TGFβ1 (BMS249-4, Invitrogen, range = 31 to 2000 pg/mL, sensitivity: 8.6 pg/mL), TGFβ2 (BMS254, Invitrogen, Waltham, MA, USA, range = 31 to 1000 pg/mL, sensitivity: 6.6 pg/mL), FGF1 (EHFGF1, Invitrogen, range = 16.38 to 4000 pg/mL, sensitivity: 12 pg/mL), and VEGF (KHG0111, Invitrogen, range = 23.4 to 1500 pg/mL, sensitivity: 5 pg/mL) were quantified using an ELISA kit according to the manufacturer’s protocol. All samples were measured twice using a Multiskan™ FC microplate photometer (Thermo Scientific, Alab, Warsaw, Poland).

### 2.4. Quantification of Soluble Collagen Using the Sircol™ Colorimetric Test

The release of soluble collagen in the culture medium incubated with A-PRF+ alone, A-PRF+ with fibroblasts, and only fibroblasts alone at days 1, 2, 3, and 7 was analyzed with the Sircol™ assay according to the manufacturer’s protocol (Biocolor Ltd., Carrickfergus, UK). The collected media were incubated with Sircol™ dye, which binds to soluble collagen, and then centrifuged to form pellets. Pellets were solubilized in sodium hydroxide, and the amount of eluted dye was measured using a Multiskan™ FC microplate photometer (Thermo Scientific, Alab, Warsaw, Poland) at 540 nm. Collagen standards supplied with the kit were used as controls.

### 2.5. Preparation of the Conditioned Media

Primary human fibroblasts at a concentration of 4 × 10^5^ cells/mL were placed into a six-well dish with 2.5 mL of culture media (DMEM) and allowed to attach. Then, the medium was replaced with a fresh one, and sterile-flattened A-PRF+ clots, obtained as described in the previous paragraph, were placed into the well and incubated for 3 days on a plate shaker at 37 °C. A-PRF+ clots without fibroblasts were also incubated for 3 days in 2.5 mL of culture media (DMEM) on a plate shaker at 37 °C. After this time, the fluid was drawn, and conditioned media containing 20% of the pooled fluid suspended in DMEM were prepared. Concurrently, fibroblasts with culture medium, as well as culture medium alone, were incubated in the same conditions and prepared as conditioned control media.

### 2.6. Cell Migration Assay

The in vitro wound healing assay for probing collective cell migration in two dimensions was performed using 2-well silicone inserts (Ibidi GmbH, Planegg, Germany) placed into a 6-well plate, which allowed the experimental variables to be standardized. To detect migration, 5 × 10^4^ cells/well were suspended in a volume of 70 μL 10% FCS/DMEM. The cell culture inserts were removed after 24 h, leaving a defined cell-free gap of 500 µm. At this time point (0 h), the fresh medium was supplemented with medium enriched with culture fluid after a 3-day incubation with A-PRF+ alone, A-PRF+ and fibroblasts, fibroblasts alone, and DMEM alone, and then placed into each well, and images were taken.

Cell cultures were observed and photographed under the CKX41 Olympus microscope (Tokyo, Japan) after 24 and 48 h. Software ImageJ (LOCI, University of Wisconsin) was used to quantify the areas of the closing gap.

### 2.7. Cell Viability Assay

HGFs were seeded into black 96-well plates. After 24 h, the fresh medium supplemented with medium enriched with culture fluid after a 3-day incubation with A-PRF+ alone, A-PRF+ and fibroblasts, fibroblasts alone, and DMEM alone was added into each well for 24, 48, and 72 h. All experiments were performed in quadruplicate. After the incubation, a PrestoBlue assay was performed to determine cell viability. The method is based on resazurin, which functions as a cell viability indicator. Viable cells convert the dark blue oxidized form of the dye (resazurin) into a red fluorescent reduced form (resorufin; λ_Ex_ = 570 nm; λ_Em_ = 590 nm).

PrestoBlue reagent (Thermo Fisher Scientific, Waltham, MA, USA) was added to each well containing 100 μL of the medium. The plate was then incubated for 30 min at 37 °C, and the change in fluorescence was measured using a Multiskan™ FC microplate photometer (Thermo Scientific, Alab, Warsaw, Poland), with the excitation/emission wavelengths set at 560/590 nm. Relative cell viability was calculated as the percentage of untreated cells.

### 2.8. Statistical Analysis

The statistical analyses of collected data (*n* = 6) were performed using Statistica version 13.3 with a significance level of α = 0.05. The normality of the distribution of variables was examined based on the Shapiro–Wilk test. The one-way analysis of variance (ANOVA) was performed for the comparison of groups’ means. ANOVA tests’ assumptions, i.e., normally distributed data, homogeneity of variance across groups, and lack of correlation between group means with variances, were controlled. In a few cases, the assumption of homogeneity of variance was found not to hold, and, therefore, for these cases, a modified ANOVA test was applied, i.e., Welch’s F-test, recommended when groups have different variances. Finally, using Tukey’s test, the post hoc analysis was performed to determine the significantly different groups. Results are presented as mean ± SD.

## 3. Results

### 3.1. Growth Factor Release from A-PRF+ Alone, A-PRF+ with Fibroblasts, and Fibroblasts Alone

**Figure 1:**
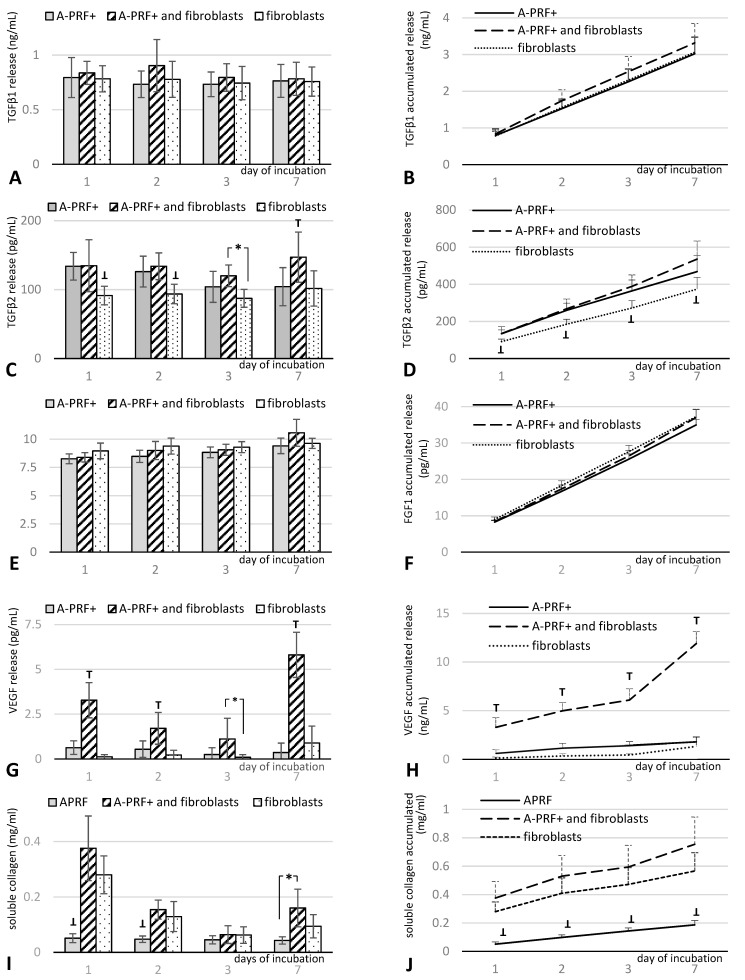
The quantification of protein released from A-PRF+ alone, A-PRF+ with fibroblasts, and fibroblasts alone at the different time points for (**A**) TGFβ1, (**C**) TGFβ2, (**E**) FGF1, (**G**) VEGF, and (**I**) soluble collagen. Total accumulated protein released over a 7-day period for (**B**) TGFβ1, (**D**) TGFβ2, (**F**) FGF1, (**H**) VEGF, and (**J**) soluble collagen. * p < 0.05, significant difference among groups; T *p* < 0.05, significantly higher than all other groups; **⊥** *p* < 0.05, significantly lower than all other groups. Data represent means ± SD from six different HGF donors.

The release of proteins, including TGFβ1, TGFβ2, FGF1, and VEGF, was quantified with ELISA, and collagen was quantified by using a spectrophotometric assay. A-PRF+ combined with fibroblasts demonstrated significantly higher values of released VEGF than both A-PRF+ alone and fibroblasts alone (Figure 1G,H), while the total release of TGFβ2 demonstrated significantly lower values for fibroblasts alone compared with A-PRF+ alone and A-PRF+ incubated with fibroblasts (Figure 1C,D). On day 7, the level of TGFβ2 was significantly higher than in the other groups (Figure 1C) and insignificantly higher after the accumulation of collected doses (Figure 1D). Moreover, the release of collagen demonstrated significantly lower values at all time points for A-PRF+ compared with A-PRF+ combined with fibroblasts and fibroblasts alone (Figure 1I,J). In comparison, no difference in the total release of TGFβ1 and FGF1 factors was observed among the three groups (Figure 1A,B,E,F).

### 3.2. Influence of Proteins Released from A-PRF+ Combined with Fibroblasts on Cell Viability

**Figure 2:**
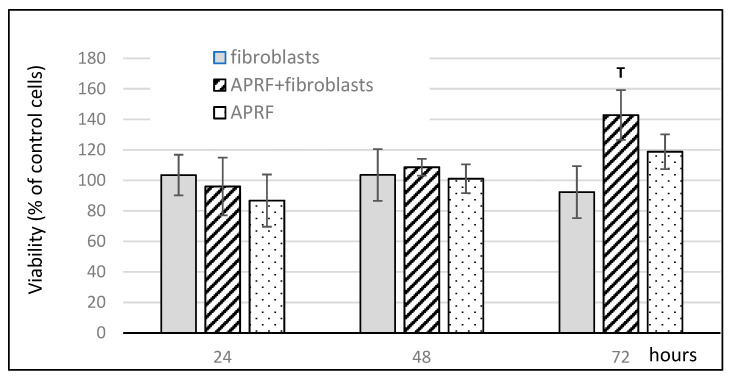
Effect of medium enriched with proteins released from A-PRF+ on fibroblast proliferation. **T** *p* < 0.05, significantly higher than all other groups. Data represent means ± SD from six different HGF donors.

The results of HGF viability after stimulation by the proteins released from A-PRF+ combined with fibroblasts are shown in Figure 2. After 72 h, there was a significant increase in cell viability after exposure to the proteins released from A-PRF+ combined with fibroblasts compared to the media conditioned with the factors released from fibroblasts alone or A-PRF+ alone. A slight decrease in cell viability was observed for the control medium conditioned with the compounds released from the fibroblasts and an increase was observed for the control medium conditioned with the proteins released from A-PRF+.

### 3.3. Enhanced Wound Healing Potential of Primary Human Gingival Fibroblasts Induced with Proteins Released from A-PRF+

**Figure 3:**
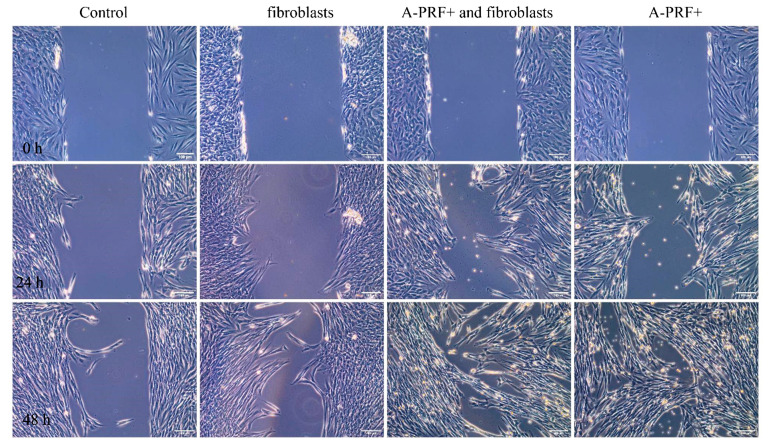
An exemplary representation of the wound healing assay under microscopic observation for control conditioned media, conditioned media with fibroblasts, with fibroblasts stimulated by A-PRF+, and with A-PRF+ alone. The scratch area is at time point 0 h, and observation time is up to 48 h.

**Figure 4:**
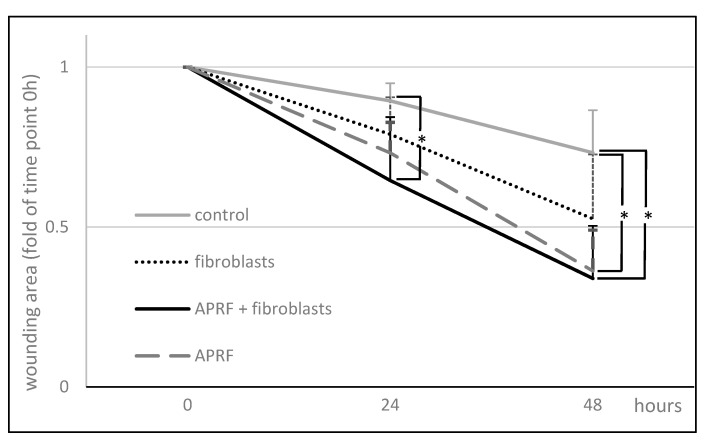
Wound closure expressed as the remaining area uncovered by the cells. The scratch area at time point 0 h was set to 48 h. * p < 0.05, significant difference among groups. Data represent means ± SD from six different HGF donors.

The effects of the factors released from fibroblasts alone, A-PRF+ alone, and fibroblasts combined with A-PRF+ on the wound healing potential of primary HGFs were analyzed by evaluating the migration of these cells using an in vitro wound healing assay. The 500 um wide gap created between the cells allowed us to analyze how the released compounds influenced the migration and invasion of cells, and the representative images of the migration of HGFs toward a wound gap are presented in Figure 3. The factors released from A-PRF+, added to the culture medium, were able to significantly increase the capacity of primary HGFs to migrate into the gap compared to controls (Figure 3 and Figure 4).

Compared to the wound area after 24 h of 11 ± 6% and 21 ± 12% for controls, which were incubated for three days either in medium alone or in medium with fibroblasts, respectively, the compounds released from A-PRF+ caused a moderate wound closure of 27 ± 10% for the factors released from A-RRF+ alone and, significantly, 35 ± 20% for the factors released from A-RRF+ combined with fibroblasts (*p* < 0.05; Figure 4). The degrees of wound closure after 48 h were significantly higher, i.e., 66 ± 16% and 64 ± 13% for the factors released from A-RRF+ alone and the factors released from A-RRF+ combined with fibroblasts, respectively, compared to 27 +/− 13% of the control wound area (*p* < 0.05; Figure 4).

## 4. Discussion

The process of soft-tissue regeneration is a cascade of signaling reactions involving the immune system; platelets; and components of connective tissue, including fibroblasts [18]. They affect blood coagulation, activating the inflammatory process, which affects migration, the proliferation of cells to the injured site, and, consequently, the remodeling of the newly created matrix [21]. In geriatric patients or individuals with immunodeficiency conditions, such as diabetes mellitus, or patients with the inability of connective tissue to proliferate and provide recession coverage, intracellular and intercellular signaling is often disturbed, and the number of cells, including fibroblasts, is reduced. The destruction of capillaries reduces ion transport. The resulting inhibition of the migration of fibroblasts from the circumferential rifer of the wound slows down the regeneration process [17,22,23]. Therefore, it is important to use biomaterials that can stimulate the host cells and, at the same time, provide the optimal amount of cells to initiate the regeneration process at the wound site. Numerous studies have shown that platelet concentrates (PCs), including PRF, promote the adhesion, proliferation, and migration of HGFs [24,25]. Steller at al. showed the crucial impact of platelet concentrates (PCs) in an effort to enhance the local treatment of bisphosphonate-related osteonecrosis of the jaw [9]. The present study demonstrates the potential of A-PRF+ with autogenous human fibroblasts as a connective tissue substitute in the augmentation of keratinized gingiva. To the authors’ knowledge, this is the first study concerning this issue. To date, the family of PRF matrices has been investigated alone, without the addition of fibroblasts [11].

The study presented in this paper compared the number of released growth factors in three groups: (1) human gingival fibroblasts alone, (2) A-PRF+ alone, and (3) A-PRF+ enriched with autologous fibroblasts. The obtained results showed a significant increase in the released VEGF in the group of A-PRF+ with autogenous human fibroblasts over a period of 7 days. One of the basic factors of proper tissue regeneration is providing nutrition through angiogenesis. The formation of a vascular network is required for the migration and proliferation of cells, which, by releasing modulators of the immune system, lead to the repopulation of the extracellular matrix and the formation of new tissue [26]. The result presented in this paper revealed a positive response in clinical terms, as according to Cabaro et al., as well as others, VEGF inhibits the hyperreactivity of T lymphocytes in the early stage of inflammation and stimulates the migration of macrophages and fibroblasts [27]. Fujioka-Kobayashi et al. showed a much higher release of VEGF from the A-PRF+ matrix up to day 3 compared to the tested LPRF and A-PRF [10]. However, from day 3 to day 10, the amount of the released VEGF was constant. Our results show that A-PRF+ enriched with autologous fibroblasts releases a statistically significantly higher amount of VEGF than that of the other groups at all points of time. It is likely that it could be the effect of stimulation by both the carrier, i.e., A-PRF+, and the fibroblasts implemented on it. In healthy patients, the formation of a wound triggers a cascade of signaling reactions involving various cells, including components of connective tissue, such as fibroblasts [18,19,20,21,22,23,24,25,26,27,28]. The activation of the inflammatory process affects the migration and proliferation of cells to the injured site and, consequently, leads to the remodeling of the newly formed extracellular matrix [21].

TGFβ is a cytokine activated by platelets in the fibrin network of the A-PRF+ matrix. It includes, among others, the TGFβ1 and TGFβ2 isoforms. It is responsible for angiogenesis, and it stimulates the chemotaxis of fibroblasts and their differentiation into myofibroblasts, which are involved in the remodeling of the extracellular matrix [29]. In the present study, an insignificant increase in TGFβ1 was obtained in the group of A-PRF+ with fibroblasts compared to the other two groups, and a significant increase in TGFβ2 in comparison to the group with fibroblasts alone. However, on day 7, the level of TGFβ2 was significantly higher than in the other groups. The described results indicate the stimulating nature of A-PRF+ on the secretion of both VEGF and TGFβ2 by fibroblasts.

Otherwise, the steady increase in the released FGF at all time points was the same in all treatment groups. FGF affects vascularization and accelerates wound healing [30,31], but not in its early stages [32]. Therefore, the results obtained in this study do not show differences between the three groups. Fibroblasts synthesize the main structural protein of type III collagen, which is replaced in the remodeling phase with type I collagen [18]. This affects the restoration of the functionality of the extracellular matrix, creating increased cross-linking of collagen fibers and, thus, increasing the stability and extensibility of collagen fibers [33]. Significantly higher values of collagen released at all time points were also observed for A-PRF+ with implanted fibroblasts compared to the A-PRF+ matrix alone. Unfortunately, the comparison with the control group of fibroblasts shows an increase but without statistical significance. This discovery confirms the reports by Masuki H. et al. in terms of the ability of the A-PRF+ matrix to induce angiogenesis and to act as a scaffold into which inter alia fibroblasts can be implemented and contribute to the acceleration of healing and subsequent regeneration of the damaged tissue [34].

Fujioka-Kobayashi et al. observed an increase in cell proliferation after exposure to A-PRF+ [10]. The present study also determined how the released components from A-PRF+ with inoculated fibroblasts affect autogenous fibroblasts. The observations up to 72 h showed a significant increase in cell viability compared to the other two test groups. The degrees of wound closure after 48 h were significant higher for the medium with the factors released from A-RRF+ alone and the factors released from A-RRF+ combined with fibroblasts in comparison to the medium with the factors released from the fibroblasts alone and from the control medium. The bioactive scaffold of the A-PRF+ matrix promotes the implementation of cells; the presented research study also shows that fibroblasts are responsible for the increased release of growth factors. Ghanaati S. et al. showed that the acquisition parameters of the A-PRF matrix are conducive to increasing its porosity [8,12]. The porosity of the carrier is important in the ability to deliver signaling cells, especially hematopoietic stem cells, for the tissue healing process [35]. This structure allows for a deeper implantation of neutrophils and, thus, their longer release. As a result, they also influence the host’s immune response at later stages of tissue healing. The finding of this study confirms the assumption that the implementation of the A-PRF+ matrix with autogenous fibroblasts could increase its clinical application.

The available data show that both the time from collection to centrifugation and the age and sex of the patient have an impact on the quality and quantity of the PRF matrix [8]. Therefore, this study aimed to show that the connection of the biomaterial with autologous cells is possible via the involvement of APRF+ with fibroblasts in wound healing, which could support recovery, especially in people whose matrix alone would be insufficient for adequate healing, e.g., in diabetic patients and in the elderly. An ideal carrier should not affect the host’s immunogenicity, and it should exhibit biocompatible properties. In turn, biodegradability should be associated with the vascularization of the recipient site and the implementation of cells, which will affect the reconstruction of the tissue defect. The used carriers with embedded signaling molecules stimulated the migration and proliferation of stem cells, thus supporting the regeneration of the target tissue. However, apart from stimulating the regeneration process, the authors would like to administrate a finished product in place of a tissue deficit. Such a solution would also accelerate regeneration in immunodeficient patients by creating bipolarity.

## 5. Conclusions

To summarize, the conducted experimental study showed a significantly increased release of VEGF and an increased viability of conditioned fibroblasts after 72 h, resulting from the combination of APRF+ and autologous human gingival fibroblasts. The obtained results indicate that the tested product, i.e., APRF+ with cultured fibroblasts, may considerably enhance the healing of surgical wounds, which is especially important in patients for whom the healing process is more problematic.

**Limitations:** Our study was carried out on a group of six patients, which was not homogeneous in terms of sex, age, and the degree of immunodeficiency. However, a group of six objects is minimal for parametric statistical evaluation. The tests performed for the starting data showed a lack of outliers at the adopted level of statistical significance. The research will be continued, considering the purposeful selection of patients for homogeneous groups. However, in the present study, despite the heterogeneity of the research group, statistically significant trends and relationships were identified, indicating improvement in fibroblast proliferation.

## Data Availability

The authors are familiar with MDPI Research Data Policies. The data that support the findings of this study are available from the corresponding author upon reasonable request.
